# The Relationship Between Blood Culture, C-reactive Protein, and Neonatal Sepsis: A Cross-Sectional Study

**DOI:** 10.7759/cureus.56813

**Published:** 2024-03-24

**Authors:** Amjed A Ali, Mustafa Ahmed, Sufian K Noor, Leena Mustafa, Waad Ibrahim, Mohamed ElAmin, Hatim M Badri, Hatim A Natto, Wahaj A Khan, Ahmed A Osman

**Affiliations:** 1 Department of Medicine, Faculty of Medicine, Nile Valley University, Khartoum, SDN; 2 Department of Medicine, Faculty of Medicine, Ahfad University for Women, Khartoum, SDN; 3 Department of Paediatrics, Faculty of Medicine, Nile Valley University, Khartoum, SDN; 4 Internal Medicine, Atbara District Hospital, Khartoum, SDN; 5 Department of Environmental and Occupational Health, College of Public Health and Health Informatics, Umm Al-Qura University, Makkah, SAU; 6 Department of Epidemiology, College of Public Health and Health Informatics, Umm Al-Qura University, Makkah, SAU; 7 Department of Preventive Medicine, Faculty of Medicine, Kassala University, Kassala, GBR

**Keywords:** pediatrics, neonatal sepsis, newborns, c-reactive protein level, blood culture

## Abstract

Background

Sepsis is one of the most common causes of morbidity and mortality in newborns. Diagnosis of neonatal sepsis may be difficult because the clinical presentations are often nonspecific. Neonatal sepsis may have an early onset (zero to three days) or a late onset (four days or later). Onset is most rapid in premature neonates. In this study, we aimed to assess the correlation between positive cultures, high C-reactive protein (CRP) levels, and the diagnosis of neonatal sepsis.

Methodology

This descriptive, prospective, cross-sectional study was undertaken over four months starting from December 15, 2019, to April 15, 2020, in Atbara Teaching Hospital, Sudan. Data were collected from 71 patients. CRP levels were measured, and blood cultures were performed.

Results

High CRP level >10 mg/L was seen in patients having positive blood culture (55.3%), mainly in preterm babies (CRP >10 mg/dL (61.1%), positive culture (55.6%)) and very low birth weight babies (CRP >10 mg/dL (83.3%) and positive culture (67%)).

Conclusions

Our findings suggest that *Klebsiella *is an important cause of neonatal sepsis. CRP was positive in babies mainly with proven sepsis. There is a high correlation between CRP and blood culture in patients with neonatal sepsis which may give access to remodeling the prioritization of the management options in the clinical setting.

## Introduction

Neonatal sepsis is used to designate a systemic condition of bacterial, viral, or fungal (yeast) origin associated with hemodynamic changes and other clinical manifestations. It results in substantial morbidity and mortality. Despite years of clinical experience in the care of neonates with confirmed or suspected sepsis, challenges remain such as the absence of a consensus definition of neonatal sepsis [1]. Neonatal sepsis is the third leading cause of neonatal mortality, only behind prematurity and intrapartum-related complications (or birth asphyxia) [2]. Every year, 2.6 million neonates die, with three-fourths of these deaths occurring in the first week of life, and almost all (99%) in low- and middle-income countries (LMICs) [3,4]. Population-level estimates of laboratory-confirmed sepsis in high-income countries are well-studied and documented; however, accurate incidence and associations remain understudied and undetermined in LMICs [5,6]. In resource-poor settings, efficient and effective diagnosis of sepsis, including identification and antibiotic susceptibility reporting, is challenging [7]. Common laboratory tests and clinical manifestations (including white blood cell indices, acute-phase reactants, and heart rate characteristics) have limited diagnostic accuracy (low positive predictive value (PPV)) for neonatal sepsis [8,9]. Although significant research has been conducted on neonatal sepsis, an accurate and rapid biomarker has not been identified yet. The increased incidence of neonatal hospital admissions to the neonatal intensive care unit (NICU) due to neonatal sepsis raises the need for finding a simple procedure for identifying neonates with sepsis, especially in developing countries as the resources are limited. This has a great public health importance. As diagnostic tests such as blood cultures are time-consuming, diagnosis is delayed and becomes problematic. Undoubtedly, early diagnosis of neonatal sepsis is essential to improving neonatal outcomes. C-reactive protein (CRP) serial measurements in infection progression are helpful and aid infection diagnosis. The negative predictive value, PPV, sensitivity, and specificity in infants (term and preterm) were observed to be 76.92%, 53.49%, 80%, and 48.94%, respectively [10].

One of the major difficulties in the management of neonatal sepsis is to reach an accurate and early diagnosis. Unlike older patients, newborns have very subtle presentations and similar conditions such as intraventricular hemorrhages and enterocolitis [11].

This study aimed to evaluate the correlation between CRP, blood culture, and neonatal sepsis in the NICU at Atbara Teaching Hospital (ATH), Sudan.

## Materials and methods

This descriptive, prospective, cross-sectional study was conducted at ATH, Sudan. ATH is situated in the western north of Atbara town, Sudan. The study was conducted from December 15, 2019, to April 15, 2020, and included neonates admitted to ATH with suspected neonatal sepsis. The inclusion criteria comprised any neonates with suspected neonatal sepsis and admitted to the hospital. The study sample included all neonates who fulfilled the inclusion criteria due to the limited number of cases. The estimated number of cases according to hospital records was 50-60 cases. We included 71 neonates to be enrolled in this study. We used a total coverage sampling technique. We drew blood samples from the patients and sent them to the laboratory to conduct a blood culture by extracting 2 mL of blood from the patient once or twice as much as possible. The nurse or technician used a standard phlebotomy technique to draw several samples of blood from the arm of the patient. Generally, we collected multiple blood samples from different veins to increase the chance of detecting any microorganisms in the bloodstream. We preferred venous blood to arterial as the veins run superficially and are easily detected. Subsequently, we submitted the blood sample to the laboratory to perform a blood culture test. This needed 1 mL of blood per sample [7].

Data collection tools and methods

We used a data collection instrument specially designed for data collection in this study, which included questions that fulfilled the objectives. The questionnaire was filled out by a researcher through a direct interview with the babies’ mothers. The first step was to obtain consent from the participants, and then the questionnaire was directly filled out. Nurses took the blood samples for CRP. In each patient, 1 mL of blood per sample was drawn and then placed in a tube pre-filled with heparin. The container was then put in a chemical analyzer biosystem A25. Samples for blood culture were taken by the researcher. For this, 1 mL of blood per sample from each patient was sent to the lab, where it was mixed with a culture to help bacteria or yeast grow if they were present in the blood. Blood cultures may yield early results within 24 hours, but it could also need 48-72 hours to determine the nature of yeast or bacteria present.

The study variables comprised sociodemographic features of the neonates as well as the CRP levels and the results of blood culture.

Data analysis

We tabulated and analyzed the data using SPSS version 21 (IBM Corp., Armonk, NY, USA). We entered data in a Microsoft Excel sheet (Microsoft Corp., Redmond, WA, USA) after coding and then transferred it to SPSS. We calculated frequencies and percentages, and the correlations were drawn using the chi-square test with a p-value to be considered significant at a confidence interval of 95%. Results were analyzed and discussed and recommendations were suggested.

Expected outcome

The study expected a relationship between CRP and blood culture in neonatal sepsis.

Ethical considerations

We obtained ethical clearance from the Sudan Medical Specialization Board (SMSB) Ethical Committee. Additionally, we obtained the required hospital permission to conduct this study. Moreover, we obtained written consent from the mothers of the babies. We considered the confidentiality of the data collected (no names were collected, and data were coded and interpreted in the form of statement tables).

## Results

We conducted our analysis among 71 neonates with suspected sepsis in a descriptive, cross-sectional, hospital-based study. The data are represented as frequency and percentages (%). Most neonates (59, 83.1%) were less than 72 hours of age, more than half were females, more than half were from Atbara City, 31 (43.7%) were from other areas, more than half were delivered vaginally, and more than two-thirds were term babies. More than half of the neonates were born with an average weight, nearly one-third had low birth weight (LBW), six (8.5%) had very low birth weight (VLBW), and five (7%) were sizable (weighing more than 4,000 g) (Table 1). The normal physical examination of the newborn included normal breathing without chest recession and no audible sounds (such as grunting), normal body temperature, normal skin texture, and color (well perfused, pink in color, and without jaundice), normal eyes without signs of infection or jaundice, normal heart rate (120 to 160 beats per minute), and the mouth with the normal sucking reflex. In this study, nearly half of the patients had abnormal heart rates, and more than one-fifth had respiratory distress (Table 1). A urine culture is important to determine any urinary tract infection in a newborn. However, in this study, we could not conduct a urine culture due to some technical and financial difficulties.

**Table 1:** Sociodemographic data and symptoms before the blood culture of the participants (N = 71). ^†††^: Sizable = babies weighing more than 4,000 g. GA = gestation age; NVD = normal vaginal delivery; EMCS = emergency cesarean section; ELCS = elective cesarean section; LBW = low birth weight; VLBW = very low birth weight; DIC = disseminated intravascular coagulation

Variable		Frequency (%)
Gender	Male	34 (47.9%)
	Female	37 (52.1%)
Age (hours)	>72	12 (16.9%)
	<72	59 (83.1%)
GA (weeks)	<37	18 (25.4%)
	37–40	52 (73.2%)
	>40	1 (1.4%)
Residence	Atbara	40 (56.3%)
	Aldamer	2 (2.8%)
	Barbar	4 (5.6%)
	Sidon	11 (15.5%)
	Others	14 (19.7%)
Mode of delivery	NVD	38 (53.5%)
	EMCS	24 (33.8%)
	ELCS	6 (8.5%)
	Assisted delivery	3 (7%)
Weight	Average	39 (54.9%)
	LBW	21 (29.6%)
	VLBW	6 (8.5%)
	Sizable	5 (7%)
Maturity	Term	49 (69%)
	Preterm	17 (24%)
	Postdate	5 (7%)
Symptoms observed before the blood culture	Abnormal heart rate	33 (46.5%)
	Respiratory distress	16 (22.54%)
	Temperature instability	26 (36.6%)
	Jaundice	13 (18.3%)
	DIC	6 (8.4%)

All study participants underwent a CRP investigation. More than half of CRP results were found to be >10 mg/L. Additionally, more than half of the cultures showed no growth. Among the 71 neonates with suspected neonatal sepsis, more than half had confirmed sepsis, 13 (18.3%) were diagnosed with sepsis plus jaundice, nine (12.7%) had hypoxic-ischemic encephalopathy plus sepsis, and more than half stayed more than five days in the nursery and received treatments. Moreover, 20 (14.1 %) were complicated with anemia and disseminated intravascular coagulation, 59 (83.1%) were discharged without complications, and eight (11.3%) died (Table 2). The data are represented as frequency and percentages (%).

**Table 2:** Diagnosis, duration of stay at NICU, CRP results, blood culture results, treatments, complications, and neonatal outcomes (N = 71). HIE + RDS = hypoxic-ischemic encephalopathy + respiratory distress syndrome; IUGR = intrauterine growth restriction; DIC = disseminated intravascular coagulation; NICU = neonatal intensive unit; CRP = C-reactive protein

Variable		Frequency (%)
CRP results among neonates	<5	23 (32.4%)
	5–10	10 (14.1%)
	>10	38 (53.5%)
Blood culture results	No results yielded	39 (54.9%)
	Klebsiella	20 (28.2%)
	Pseudomonas aeruginosa	5 (7%)
	Staphylococcus aureus	4 (5.6%)
	Escherichia coli	3 (4.2%)
Diagnosis	Sepsis	39 (54.9%)
	Sepsis + HIE + RDS	4 (5.6%)
	Sepsis + HIE	9 (12.7%)
	Sepsis + RDS	4 (5.6%)
	Sepsis + IUGR	2 (2.8%)
	Sepsis + Jaundice	13 (18.3%)
Duration of stay at the NICU (days)	>5	40 (56.3%)
	<5	31 (43.7%)
Treatments	Received oxygen	18 (25.4%)
	Received phototherapy	23 (32.4%)
	Received blood	8 (11.2%)
	Received anticonvulsant drugs	6 (8.5%)
	Received fluid + electrolytes	24 (33.8%)
	Received antibiotics	71 (100%)
Complications and outcomes	Discharged without complications	59 (83.1%)
	Discharged with complications	1 (1.4%)
	Death	8 (11.3%)
	Anemia	4 (5.6%)
	DIC	6 (8.5%)

CRP levels were higher among patients aged less than 72 hours in 31 patients in comparison to patients aged more than 72 hours in seven patients but with no statistical significance (Table 3). The data are represented as frequency and percentages (%), and the p-value is considered significant at <0.05.

**Table 3:** Age and CRP correlations showing insignificant statistical relations (N = 71). CRP = C-reactive protein

Age	CRP			Total	P-value
	<5	5–10	>10		
<72 hours	19 (32.2%)	9 (15.3%)	31 (52.5%)	59 (100%)	0.873
>72 hours	4 (33.3%)	1 (8.3%)	7 (58.3%)	12 (100%)	
Total	23 (32.4%)	10 (14.1%)	38 (53.5%)	71 (100%)	

The data showed that most patients were aged less than 72 hours and were discharged without complication, while seven (11.9%) died, with no statistical significance. Female patients were discharged more often without complication in comparison to male patients but without statistical significance. With a statistical significance, the CRP was higher (more than 10 mg/dL) among patients who died (8, 21.1%). *Klebsiella *was the most commonly detected organism affecting patients and led to the death of five (25%) neonates with no statistical significance (Table 4). The data are represented as frequency and percentages (%), with the p-value considered significant at <0.05.

**Table 4:** The association between neonatal sepsis outcomes and age, sex, CRP, and blood culture (N = 71). DWNC = discharge with no complications; DWC = discharge with complications; DAMA = discharge against medical advice; CRP = C-reactive protein

Variables		Neonatal sepsis outcome				Total	P-value
		DWNC	DWC	DAMA	Death		
Age (hours)	<72	51 (86.4%)	1 (1.7%)	0 (0%)	7 (11.9%)	59 (100%)	0.24
	>72	8 (66.7%)	0 (0%)	3 (25%)	1 (8.3%)	12 (100%)	
	Total	59 (83.1%)	1 (1.4%)	3 (4.2%)	8 (11.3%)	71 (100%)	
Sex	Male	27 (79.4%)	1 (2.9%)	2 (5.9%)	4 (11.8%)	34 (100%)	0.48
	Female	32 (86.5%)	0 (0%)	1 (2.7%)	4 (10.8%)	37 (100%)	
	Total	59 (83.1%)	1 (1.4%)	3 (4.2%)	8 (11.3%)	71 (100%)	
CRP (mg/L)	5	21 (91.3%)	0 (0%)	2 (8.7%)	0 (0%)	23 (100%)	0.03
	5–10	10 (100%)	0 (0%)	0 (0%)	0 (0%)	10 (100%)	
	>10	28 (73.7%)	1 (2.6%)	1 (2.6%)	8 (21.1%)	38 (100%)	
	Total	59 (83.1%)	1 (1.4%)	3 (4.2%)	8 (11.3%)	71 (100%)	
Blood culture	No growth	34 (87.2%)	1 (2.6%)	1 (2.6%)	3 (7.7%)	39 (100%)	0.77
	Klebsiella	14 (70%)	0 (0%)	1 (5%)	5 (25%)	20 (100%)	
	Pseudomonas	4 (100%)	0 (0%)	0 (0%)	0 (0%)	4 (100%)	
	Escherichia coli	1 (50%)	0 (0%)	1 (50%)	0 (0%)	2 (100%)	
	Staphylococcus aureus	4 (100%)	0 (0%)	0 (0%)	0 (0%)	4 (100%)	
	Others	2 (100%)	0 (0%)	0 (0%)	0 (0%)	2 (100%)	
	Total	59 (83.1%)	1 (1.4%)	3 (4.2%)	8 (11.3%)	71 (100%)	

Regarding the association between sepsis accompanied by jaundice and neonatal sepsis complication versus age, sex, CRP, and blood culture, we found that sepsis accompanied by jaundice affected a considerable number of patients. It was higher in patients aged less than 72 hours in comparison to patients aged more than 72 hours (21 (35.6%) versus 2 (16.7%)) but without statistical significance, female patients (8 (23.5%) versus 15 (40.5%), higher CRP (more than 10) (12 (31.6%) versus 7 (30.4%)) but without statistical significance, and in patients with positive *Klebsiella *results with statistical significance (Table 5). The data are represented as frequency and percentages (%), with a p-value <0.05 considered significant.

**Table 5:** The association between the presence of jaundice and complications (anemia and DIC) versus other factors (age, sex, CRP, and blood culture) (N = 71). DIC = disseminated intravascular coagulation; CRP = C-reactive protein

Variables		Presence of jaundice and complications of neonatal sepsis					Total	P-value
		Jaundice	Anemia	DIC	Other	None		
Age (hours)	<72	21 (35.6%)	3 (5.1%)	2 (3.4%)	2 (3.4%)	31 (52.5%)	59 (100%)	0.442
	>72	2 (16.7%)	1 (8.3%)	0 (0%)	2 (16.7%)	7 (58.3%)	12 (100%)	
	Total	23 (32.4%)	4 (5.6%)	2 (2.8%)	4 (5.6%)	38 (53.5%)	71 (100%)	
Sex	Male	8 (23.5%)	1 (2.9%)	2 (5.9%)	4 (11.8%)	19 (55.9%)	34 (100%)	0.18
	Female	15 (40.5%)	3 (8.1%)	0 (0%)	0 (0%)	19 (51.4%)	37 (100%)	
	Total	23 (32.4%)	4 (5.6%)	2 (2.8%)	4 (5.6%)	38 (53.5%)	71 (100%)	
CRP	<5	7 (30.4%)	1 (4.3%)	1 (4.3%)	1 (4.3%)	13 (56.5%)	23 (100%)	0.64
	5–10	4 (40%)	0 (0%)	0 (0%)	0 (0%)	6 (60%)	10 (100%)	
	>10	12 (31.6%)	3 (7.9%)	1 (2.6%)	3 (7.9%)	19 (50%)	38 (100%)	
	Total	23 (32.4%)	4 (5.6%)	2 (2.8%)	4 (5.6%)	38 (53.5%)	71 (100%)	
Blood culture	No growth	13 (33.3%)	2 (5.1%)	2 (5.1%)	1 (2.6%)	21 (53.8%)	39 (100%)	0.05
	Klebsiella	7 (35%)	2 (10%)	0 (0%)	3 (15%)	8 (40%)	20 (100%)	
	Pseudomonas	3 (75%)	0 (0%)	0 (0%)	0 (0%)	1 (25%)	4 (100%)	
	Escherichia coli	0 (0%)	0 (0%)	0 (0%)	0 (0%)	2 (100%)	2 (100%)	
	Staphylococcus aureus	0 (0%)	0 (0%)	0 (0%)	0 (0%)	4 (100%)	4 (100%)	
	Others	0 (0%)	0 (0%)	0 (0%)	0 (0%)	2 (100%)	2 (100%)	
	Total	23 (32.4%)	4 (5.6%)	2 (2.8%)	4 (5.6%)	38 (53.5%)	71 (100%)	

## Discussion

Despite significant advancements in neonatal care and extensive research efforts in developed countries, the grim reality persists: four out of every 10 infants afflicted with sepsis either succumb to the condition or suffer substantial disabilities, including enduring neurodevelopmental impairments [12]. This distressing statistic could potentially be even more dire in LMICs. Therefore, there is an urgent and pressing need to redirect research efforts toward tackling neonatal sepsis in LMICs. Such a shift in focus is crucial to advancing the third goal outlined by the World Health Organization (WHO), which aims to reduce newborn and child mortality by 2030, ultimately putting an end to preventable deaths among newborns and children under the age of five years.

A cohort of 71 infants diagnosed with sepsis was admitted for assessment. Among these cases, 32 (45%) infants were confirmed to have sepsis based on both positive blood culture results and clinical signs indicative of sepsis. For the remaining 39 (54.9%) infants, a diagnosis of probable sepsis was made, as their blood cultures yielded negative results, but they exhibited clinical symptoms consistent with sepsis.

The CRP assay yielded noteworthy findings, with positive results obtained in 40 (55.3%) patients with confirmed sepsis and 31 (44.7%) patients with probable sepsis. Notably, the difference in CRP positivity between these two groups was statistically significant (p = 0.020). Previous studies have highlighted various factors that are linked to an elevated risk of neonatal sepsis. These factors include LBW, preterm birth (occurring before 37 weeks of gestation), premature rupture of membranes, neonatal sex, complications during the intrapartum period such as perinatal asphyxia, low socioeconomic status, inadequate sanitation conditions, malnutrition, and overcrowding [13-15].

Traditionally, the detection of a positive blood culture has been considered the gold standard for diagnosing neonatal sepsis [16].

In our study, we observed that a high CRP level exceeding 10 mg/L was notably prevalent among cases with positive blood culture results, constituting 41 (55.3%) of the affected infants. This trend was particularly prominent among preterm infants, where a CRP level exceeding 10 mg/L was identified in 43 (61.1%) cases, and among VLBW infants (LBW: 21 (29.6%), and VLBW: 6 (8.5%)), with 59 (83.3%) exhibiting elevated CRP levels, and 47 (67%) having positive blood culture results. We observed that a high CRP level (>10 mg/L) was prevalent in term babies at 37 (51.9%), while early preterm babies exhibited an even higher rate at 43 (61.1%). Our findings align with the study reported by Desai et al., which also identified significantly elevated CRP levels in preterm neonates with positive blood cultures when compared to term babies [16]. However, this contradicts the results of a previous study, which found a significant correlation between CRP levels and maturity, particularly in neonates at 33 weeks of gestational age (GA) versus 29 weeks of GA. Additionally, another study reported an association between GA and the magnitude of clinically relevant CRP response, with a lower response of six (8%) in newborns aged 24-27 weeks compared to those born between 40 and 41 weeks at 18 (25%). Moreover, Kawamura et al. reported a lower sensitivity of CRP in diagnosing neonatal sepsis in preterm newborns compared to term babies (61.5% vs. 75%), challenging the idea that the baby’s maturity reflects the maturation of the immune system, as suggested by Brankica [10].

Our study revealed that *Klebsiella *was the most commonly isolated organism, accounting for 20 (28%) cases, followed by *Staphylococcus aureus*. This is consistent with the findings of a previous study, where *Klebsiella *was also the most prevalent organism, followed by *Staphylococcus aureus*. Interestingly, in term babies, *Klebsiella *was the predominant isolated organism at 26.9%, contradicting the findings of Desai et al., which identified *Streptococcus *as the primary pathogen at 75%. In preterm babies, our study demonstrated that *Klebsiella *was commonly isolated (33.3%), aligning with the findings of Desai et al. (67%).

We also observed that high CRP levels (>10 mg/L) were more prevalent in VLBW babies at 60 (83.3%) compared to 35 (48.7%) in average-weight babies. This differs from the study by Dritsakou et al., which found the highest CRP levels in babies weighing >2,500 g. This variation may be due to the influence of the infecting organism (Gram-negative), which commonly affects LBW infants.

Furthermore, in our study, higher CRP levels (>10 mg/L) were more frequently seen in cases with positive blood cultures (40, 55.3%) compared to those with negative blood cultures (32, 44.7%), aligning with Binzhou et al.’s findings, where positive blood cultures were associated with CRP levels >0.8 mg/dL in 39.1% of cases, while only 20.6% of those with negative blood cultures had CRP levels exceeding 0.8 mg/dL.

Moreover, similar research has been conducted in patients with inflammatory diseases of the head and neck and yielded that the delta neutrophil index can be used with other inflammatory indicators such as leukocyte concentration, CRP, and neutrophils in the peripheral blood of patients [17-20].

Limitations of the study

One of the limitations of this study is the relatively small sample size which may be attributed to the rare occurrence of neonatal sepsis cases in hospitals. Furthermore, the sensitivity results were not presented in this study due to incomplete data. Moreover, causality cannot be detected using such study designs. Follow-up and/or prospective studies are recommended.

## Conclusions

Our findings suggest that *Klebsiella *is an important cause of neonatal sepsis. CRP was positive in babies mainly with proven sepsis. The study proved that there is a high correlation between CRP and blood culture in patients with neonatal sepsis which may give access to remodeling the prioritization of the management options in the clinical setting.

## References

[1] Wynn JL, Wong HR, Shanley TP, Bizzarro MJ, Saiman L, Polin RA (2014). Time for a neonatal-specific consensus definition for sepsis. Pediatr Crit Care Med.

[2] Liu L, Johnson HL, Cousens S (2012). Global, regional, and national causes of child mortality: an updated systematic analysis for 2010 with time trends since 2000. Lancet.

[3] Wang H, Liddell CA, Coates MM (2014). Global, regional, and national levels of neonatal, infant, and under-5 mortality during 1990-2013: a systematic analysis for the Global Burden of Disease Study 2013. Lancet.

[4] Lawn JE, Cousens S, Zupan J (2005). 4 million neonatal deaths: when? Where? Why?. Lancet.

[5] Rudd KE, Kissoon N, Limmathurotsakul D (2018). The global burden of sepsis: barriers and potential solutions. Crit Care.

[6] Taylor AW, Blau DM, Bassat Q (2020). Initial findings from a novel population-based child mortality surveillance approach: a descriptive study. Lancet Glob Health.

[7] Ullah O, Khan A, Ambreen A, Ahmad I, Akhtar T, Gandapor AJ, Khan AM (2024). Antibiotic sensitivity pattern of bacterial isolates of neonatal septicemia in Peshawar, Pakistan. Arch Iran Med.

[8] Benitz WE (2010). Adjunct laboratory tests in the diagnosis of early-onset neonatal sepsis. Clin Perinatol.

[9] Coggins SA, Weitkamp JH, Grunwald L, Stark AR, Reese J, Walsh W, Wynn JL (2016). Heart rate characteristic index monitoring for bloodstream infection in an NICU: a 3-year experience. Arch Dis Child Fetal Neonatal Ed.

[10] Kawamura M, Nishida H (1995). The usefulness of serial C-reactive protein measurement in managing neonatal infection. Acta Paediatr.

[11] Camacho-Gonzalez A, Spearman PW, Stoll BJ (2013). Neonatal infectious diseases: evaluation of neonatal sepsis. Pediatr Clin North Am.

[12] Brocklehurst P, Farrell B, King A (2011). Treatment of neonatal sepsis with intravenous immune globulin. N Engl J Med.

[13] Murthy S, Godinho MA, Guddattu V, Lewis LE, Nair NS (2019). Risk factors of neonatal sepsis in India: a systematic review and meta-analysis. PLoS One.

[14] (2021). WHO. Newborn death and illness. http://www.who.int/pmnch/media/press_materials/fs/fs_newborndeath_illness/en/Date:2011.

[15] Bohanon FJ, Nunez Lopez O, Adhikari D, Mehta HB, Rojas-Khalil Y, Bowen-Jallow KA, Radhakrishnan RS (2018). Race, income and insurance status affect neonatal sepsis mortality and healthcare resource utilization. Pediatr Infect Dis J.

[16] Desai PK, Singh A, Panda S, Olivas G (2019). Role of high sensitivity CRP (HS-CRP) in evaluation of preterm and term neonatal sepsis with maternal chorioamnionitis - retrospective-cohort study. Pediatrics.

[17] Yankov YG (2023). Delta neutrophil index as a new marker of purulent inflammation in men with non-odontogenic abscesses of the neck. Cureus.

[18] Yankov YG, Bocheva YD (2022). Procalcitonin and C-reactive protein in the surgery of inflammatory head and neck diseases - diagnostic significance and correlations. Varna Med Forum.

[19] Yankov YG, Bocheva YD (2022). The value of the Delta Neutrophil Index in odontogenic and non-odontogenic abscess surgery of the head and the neck. Varna Med Forum.

[20] Yankov YG, Bocheva Y (2023). Comparative characterization of procalcitonin (sensitivity, specificity, predictability, and cut-off reference values) as a marker of inflammation in odontogenic abscesses of the head and neck in the female population. Cureus.

